# Evaluation of pancreatic cancer cell migration with multiple parameters in vitro by using an optical real-time cell mobility assay device

**DOI:** 10.1186/s12885-017-3218-4

**Published:** 2017-03-31

**Authors:** Akira Yamauchi, Masahiro Yamamura, Naoki Katase, Masumi Itadani, Naoko Okada, Kayoko Kobiki, Masafumi Nakamura, Yoshiyuki Yamaguchi, Futoshi Kuribayashi

**Affiliations:** 1 Department of Biochemistry, Kawasaki Medical School, 577 Matsushima, Kurashiki, Okayama, 701-0192 Japan; 2 Department of Clinical Oncology, Kawasaki Medical School, Okayama, Japan; 3 Department of Molecular and Developmental Biology, Kawasaki Medical School, Okayama, Japan; 4 Department of Surgery and Oncology, Graduate School of Medical Sciences, Kyushu University, Fukuoka, Japan

**Keywords:** Migration, Chemotaxis, Lipid mediator, Inhibitor, TAXIScan, Metastasis

## Abstract

**Background:**

Migration of cancer cell correlates with distant metastasis and local invasion, which are good targets for cancer treatment. An optically accessible device “TAXIScan” was developed, which provides considerably more information regarding the cellular dynamics and less quantity of samples than do the existing methods. Here, we report the establishment of a system to analyze the nature of pancreatic cancer cells using TAXIScan and we evaluated lysophosphatidic acid (LPA)-elicited pancreatic cell migration.

**Methods:**

Pancreatic cancer cell lines, BxPC3, PANC-1, AsPC1, and MIAPaCa-2, were analyzed for adhesion as well as migration towards LPA by TAXIScan using parameters such as velocity and directionality or for the number of migrated cells by the Boyden chamber methods. To confirm that the migration was initiated by LPA, the expression of LPA receptors and activation of intracellular signal transductions were examined by quantitative reverse transcriptase polymerase reaction and western blotting.

**Results:**

Scaffold coating was necessary for the adhesion of pancreatic cancer cells, and collagen I and Matrigel were found to be good scaffolds. BxPC3 and PANC-1 cells clearly migrated towards the concentration gradient formed by injecting 1 μL LPA, which was abrogated by pre-treatment with LPA inhibitor, Ki16425 (IC_50_ for the directionality ≈ 1.86 μM). The LPA dependent migration was further confirmed by mRNA and protein expression of LPA receptors as well as phosphorylation of signaling molecules. LPA_1_ mRNA was highest among the 6 receptors, and LPA_1_, LPA_2_ and LPA_3_ proteins were detected in BxPC3 and PANC-1 cells. Phosphorylation of Akt (Thr308 and Ser473) and p42/44MAPK in BxPC3 and PANC-1 cells was observed after LPA stimulation, which was clearly inhibited by pre-treatment with a compound Ki16425.

**Conclusions:**

We established a novel pancreatic cancer cell migration assay system using TAXIScan. This assay device provides multiple information on migrating cells simultaneously, such as their morphology, directionality, and velocity, with a small volume of sample and can be a powerful tool for analyzing the nature of cancer cells and for identifying new factors that affect cell functions.

**Electronic supplementary material:**

The online version of this article (doi:10.1186/s12885-017-3218-4) contains supplementary material, which is available to authorized users.

## Background

Migration of cancer cells correlates with distant metastasis and local invasion. This phenomenon involves various molecules including chemoattractants, trophic growth factors and their receptors, adhesion molecules, intracellular signaling molecules, motor proteins, and the cytoskeleton [1]. These molecules are orchestrated to help cells migrate to specific parts of the body or even spontaneously without an apparent destination. As cancer metastasis is directly associated with prognosis, controlling cancer cell migration is an effective strategy for treating the disease. Pancreatic cancer is among those with the poorest prognosis [2]. The treatment for this type of cancer is currently restricted as there are few effective drugs and knowledge regarding the nature of this cancer type is insufficient. New insights regarding this cancer and novel approaches for its treatment have long been awaited.

Lysophosphatidic acid (LPA) is a highly bioactive lipid mediator and is known to be involved in cancer cell migration, proliferation, and production of angiogenic factors [3]. In the process of cell migration, LPA works as a potent chemoattractant for various kinds of cells. Six receptors of LPA (LPA_1_, LPA_2_, LPA_3_, LPA_4_, LPA_5_, and LPA_6_) are known and all of them are G-protein coupled [4–9]. Some cells express one of these receptors, while others express multiple receptors for LPA [10]. Several articles have reported that pancreatic cancer cell lines express LPA receptors and the cells migrate towards LPA, using Boyden chamber and/or Transwell culture methods, which involve counting the number of migrated cells [11–13].

TAXIScan is an assay device for studying cell dynamics in vitro and has been used in the analysis of both suspension (mostly hematopoietic) and adherent cells [14–22]. The device functions as an optically accessible system and provides two-dimensional images of cell migration. TAXIScan provides markedly more information including morphology as well as quantitative analysis compared to existing methods such as Boyden chamber method. This device consists of an etched silicon substrate and a flat glass plate, both of which form horizontal channels each with a micrometer-order depth and forms 2 compartments on either side of a channel. Cells are placed and aligned on one side, while a stimulating factor is injected to the other side (typically 1 μL each of the cells and the stimulant). The cells react to the stable concentration gradient of the stimulant inside the horizontal channel [14]. The cell images are observed thereafter and filmed with a charge-coupled device camera located beneath the glass. By analyzing the cell images, many parameters can be determined including velocity, directionality, etc. [23–26].

The objective of this study is to establish TAXIScan as a system for pancreatic cancer research by using pancreatic cancer cell lines and to evaluate cancer cell migration in vitro for understanding the characteristics of this cancer cell type and for identifying new drugs to regulate cancer cell migration. Here, we show the adherence of cells to the scaffolds as well as LPA-elicited migration by TAXIScan, and by an existing method, the modified Boyden chamber method (Transwell). The LPA-elicited migration was confirmed by checking the expression of LPA receptors and the effect of an LPA inhibitor Ki16425.

## Methods

### Reagents

Fetal bovine serum (FBS) was obtained from Nichirei Biosciences Inc. (Tokyo, Japan); RPMI1640 and D-MEM were from Sigma-Aldrich (St. Louis, MO, USA); Collagen I, Matrigel (growth factor reduced), fibronectin, laminin, and collagen I pre-coated coverslips were obtained from Becton Dickinson (San Jose, CA, USA); fatty-acid-free bovine serum albumin (BSA) from Nacalai Tesque (Kyoto, Japan); LPA from Enzo Life Sciences Inc. (Farmingdale, NY, USA); Opti-MEM from Thermo Fisher Scientific Inc. (Waltham, MA, USA); Anti- LPA_1_, LPA_3_, LPA_5_, and LPA_6_ rabbit polyclonal antibodies from GeneTex Inc. (Irvine, CA, USA); anti-LPA _2_ rabbit polyclonal antibody from Abgent (San Diego, CA, USA); and anti-LPA_4_ rabbit polyclonal antibody from Acris Antibodies Inc. (San Diego, CA, USA); Ki16425 was purchased from Cayman Chemical (Ann Arbor, MI, USA).

### Maintenance of cells

Human pancreatic cancer cell lines BxPC3 (ATCC CRL-1687), PANC-1 (ATCC CRL-1469), and AsPC1 (ATCC CRL-1682) were obtained from the American Type Culture Collection (ATCC), and MIAPaCa-2 (RCB2094) and KATOIII (RCB2088) from Riken Cell Bank. PC3 and 211H were kindly provided by Dr. Masakiyo Sakaguchi. Cells were cultured and maintained in RPMI1640 with 10% FBS or in D-MEM with 10% FBS on 10-cm diameter dishes as the standard procedure. Passaging of the cells was performed using PBS and Trypsin/EDTA solution when they were 80-90% confluent. All samples were handled according to the Declaration of Helsinki.

### Migration assay

The Real-time cell mobility assay was performed by optical real-time cell mobility assay device “EZ-TAXIScan” (ECI, Inc., Kawasaki, Japan) as described previously [20], except for assembling the TAXIScan holder together with a coverslip pre-coated with the extracellular matrix. Briefly, coverslips were coated with collagen I (100 μg/mL), Matrigel (1/30 diluted solution with culture medium), fibronectin (100 μg/mL), laminin (100 μg/mL), or the culture medium, by incubating 100 μL of each solution on a coverslip at room temperature for 1 h before assembling the TAXIScan holder. After collagen I was selected as the scaffold, collagen I pre-coated coverslips were used for the TAXIScan method. The pre-coated coverslip was washed once with 0.5 mL of PBS and was placed on the glass plate for TAXIScan. The TAXIScan holder was assembled according to the manufacturer’s instructions. Cells were harvested by detaching from culture flasks using the same conditions as passaging. One μL of suspension prepared in the culture medium containing 2 × 10^6^ cells/mL was applied to the cell-injection side of TAXIScan holder and the cells (100 or less in most of the cases) were aligned at the edge of the micro-channel. After obtaining the first round of images, 1 μL of the chemoattractant solution prepared in the chemotaxis buffer was added to the ligand-injection side of the device to initiate migration. The assay conditions were as follows: duration, 4 h; interval, 5 min; micro-channel depth, 10 μm; and temperature, 37 °C. Time-lapse images of cell migration were stored as electronic files on a computer hard disk and analyzed when needed. The morphologies of migrating cells were depicted by tracing the edge of cells and then superimposing the resulting outlines onto the initial image. Movies of the images were made and quantification of velocity and directionality was carried out through the “TAXIScan analyzer 2” software. The trajectory of each cell on the image was traced by clicking the center portion of each cell on the computer display. The velocity (V) and the directionality (D) of each cell were calculated using the traced data as described previously [20, 23]. The statistical analysis for the velocity and the directionality was done by the Kruskal-Wallis Test (Non-parametric ANOVA) followed by the Dunn’s Multiple Comparisons Test, as the data did not show normal distribution in most cases [20].

The modified Boyden chamber method was performed using collagen I-coated polycarbonate membrane inserts (8 μm pore size) in a 24-well plate (CytoSelect 24-Well Cell Haptotaxis Assay kit, Cell Biolabs, Inc. San Diego, CA, USA) or Transwell Plate with non-coated polycarbonate membrane (Corning Incorporated, Corning, NY, USA), per the manufacturer’s protocols. Briefly, the cells grown on a culture dish were detached with Trypsin/EDTA solution, washed with PBS, and re-suspended in RPMI1640/HEPES buffer with 0.1% fatty-acid-free BSA (the chemotaxis buffer) to attain a density of 0.5 × 10^6^ cells/mL. A total of 1.5 × 10^5^ cells per well were placed in the upper chamber; the chemotaxis buffer with or without LPA was injected to the lower chamber, and then the plate was incubated at 37 °C for 2 h. The migrated cells were stained with the staining solution (supplied with the kit), observed under the microscope, and then lysed with the lysis solution (supplied with the kit) to quantify the number of migrated cells by measuring the absorbance at 560 nm. The absorbance was calibrated with the numbers of cells by using the standard curve with a series of different cell numbers (0, 10, 32, 100, 320, 1000, 3200, and 10,000 cells).

### Quantitative reverse transcriptase polymerase reaction (qRT-PCR)

Total RNA was extracted from the cells using the RNeasy kit (QIAGEN, Hilden, Germany). Cells were seeded on 10 cm-diameter dishes until 80-90% confluency was attained. On the day of the experiment, the medium was removed, and the cells were washed with 5 mL PBS, followed by addition of lysis solution, per the manufacture’s recommended procedure. Template DNA was prepared with extracted total RNA of each sample using Ready-To-Go You-Prime First-Strand Beads kit (GE Healthcare, Little Chalfont, UK) and 0.5 μL each of 1st strand DNA per sample was used for quantitative polymerase reaction (qPCR) with Fast SYBR Green Master Mix reagent (Life Technologies, Carlsbad, CA, USA). Analysis was done after preparing samples in a 96-well plate; signal during PCR was detected by Step One Plus Real-time PCR system (Life Technologies). The primers used are given in Additional file 1: Table S1. β-actin was used as an internal control for normalization of data. Data were analyzed by the software accompanied with the PCR system.

### Protein expression and phosphorylation detection

Cells were seeded on 10-cm-diameter dishes until 80-90% confluency was attained. On the day of the experiment, cells were rinsed once with 5 mL of serum free Opti-MEM and then stimulated with 1 μM LPA prepared in the chemotaxis assay buffer (0.1%BSA in RPMI1640) pre-warmed at 37 °C for 30 s, 2 min, or 5 min. Immediately after stimulation, the medium was replaced with ice-cold chemotaxis assay buffer and cells were kept on ice until lysis was done. Cells were lysed with ice-cold lysis buffer from the PathScan RTK Signaling Antibody Array kit (Cell Signaling Technology, Danvers, MA, USA) per the manufacture’s procedure. Cell lysate was kept at −70 °C until the PathScan phosphorylation array or SDS-PAGE/western blotting was performed. For western blotting, each cell lysate was subjected to SDS-PAGE, blotting, and antibody reaction. The pre-stained protein marker (Bio-Rad, Hercules, CA, USA) or the CruzMarker protein marker (Santa Cruz Biotechnology, Santa Cruz, CA, USA) was used to estimate the molecular weight of probed bands. Protein bands were visualized with ECL prime (GE Healthcare) and detected by LAS-4000 mini device (GE Healthcare). The list of the phosphorylated proteins for the array is shown in Additional file 2: Table S2.

## Results

### Establishing the optical real-time migration assay system for pancreatic cancer cells

**Fig. 1:**
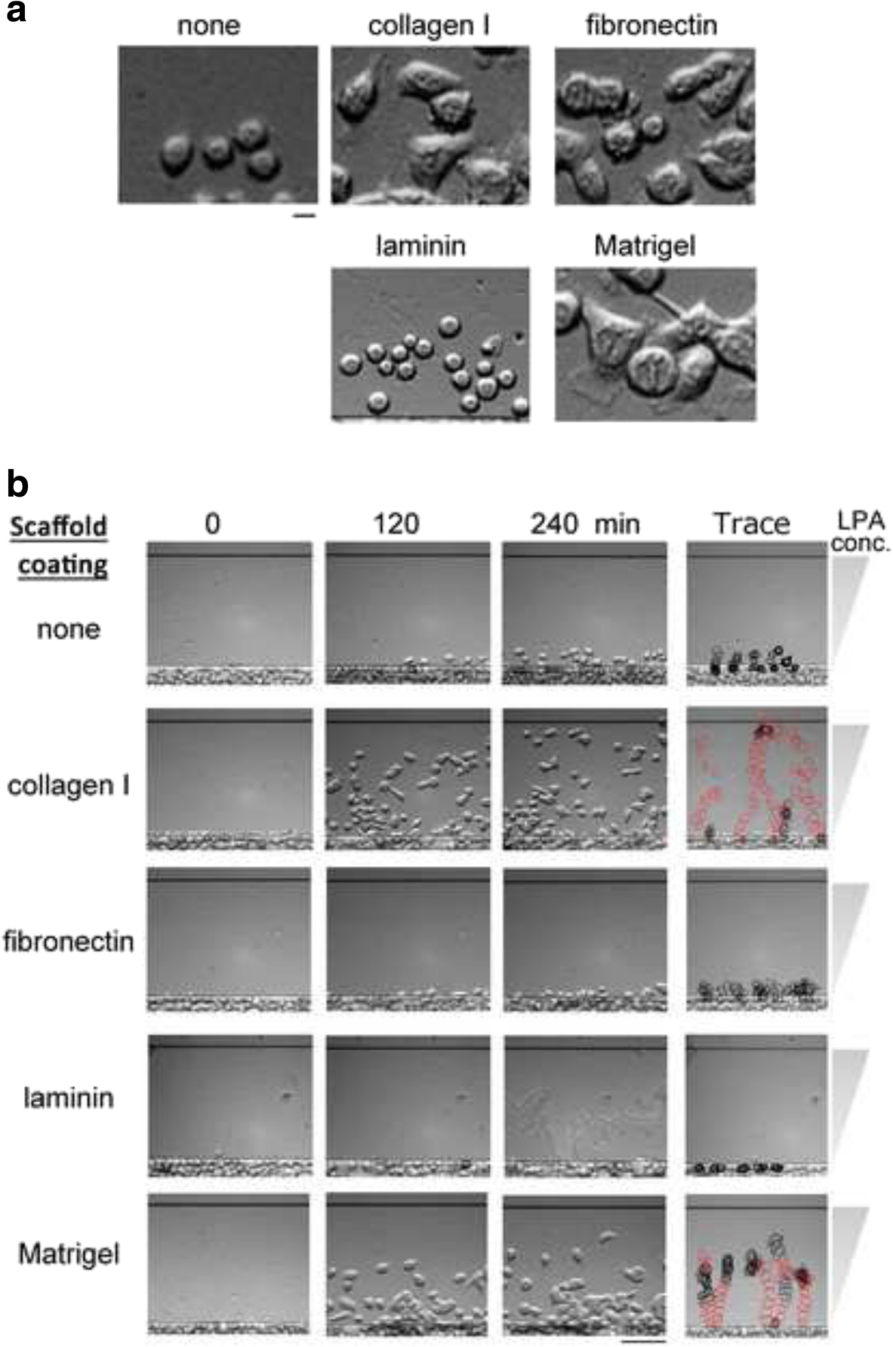
Adhesion and migration of pancreatic cancer cells monitored by TAXIScan. **a** Morphology of BxPC3 pancreatic cancer cells after adherence to each scaffold material coated on the coverslip without chemoattractant. Images were taken 240 min after starting the assay. Scale bar: 10 μm. **b** Chemotaxis of BxPC3 pancreatic cancer cells towards 100 nM LPA with or without various kinds of scaffold-coating. Images taken at time 0, 120 and 240 min are shown. The morphologies of 4 or 5 representative migrating cells throughout the assays are shown on the “Trace” column. The outlines of the migrating cells were traced every 10 min in this column. Cells migrating at more than 1 μm/min are shown in *red*. All data are representative of 3 independent experiments. Scale bar: 100 μm



We established the assay system for pancreatic cells using optically accessible horizontal cell mobility assay device, EZ-TAXIScan. This device has been used for monitoring chemotaxis assays mostly for hematopoietic cells such as neutrophils, monocytes/macrophages, dendritic cells, eosinophils, and lymphocytes [14–25]. In the case of adherent cells, like the cancer cells, additional procedures may be required for retrieving the optimal response from cells, such as scaffold coating [26]. Therefore, we compared different coatings on glass for facilitating pancreatic cell migration. Human collagen I, fibronectin, laminin, and Matrigel (growth factor reduced) were examined as scaffold substances coated on the glass plate inside the TAXIScan chamber. Among these materials, collagen I and Matrigel showed good performances (Fig. 1) (An additional movie file shows this in more detail [see Additional file 3]). Without coating, the cells did not attach well onto the glass plate (Fig. 1a) and did not show good migration (Fig. 1b). On the glass coated with collagen I or Matrigel, most cells attached and spread well even without a stimulant such as the chemoattractant (Fig. 1a). On the glass coated with collagen I or Matrigel, BxPC3 cells migrated towards LPA (Fig. 1b).

**Additional file 3:** Chemotaxis of BxPC3 pancreatic cancer cells towards 100 nM LPA with or without various kinds of scaffold-coating. Images were taken every 5 min for 4 h and movies were created by TAXIScan Analyzer2 software. Representative of 3 independent experiments. Scale bar: 100 μm. (MP4 9312 kb)


**Fig. 2:**
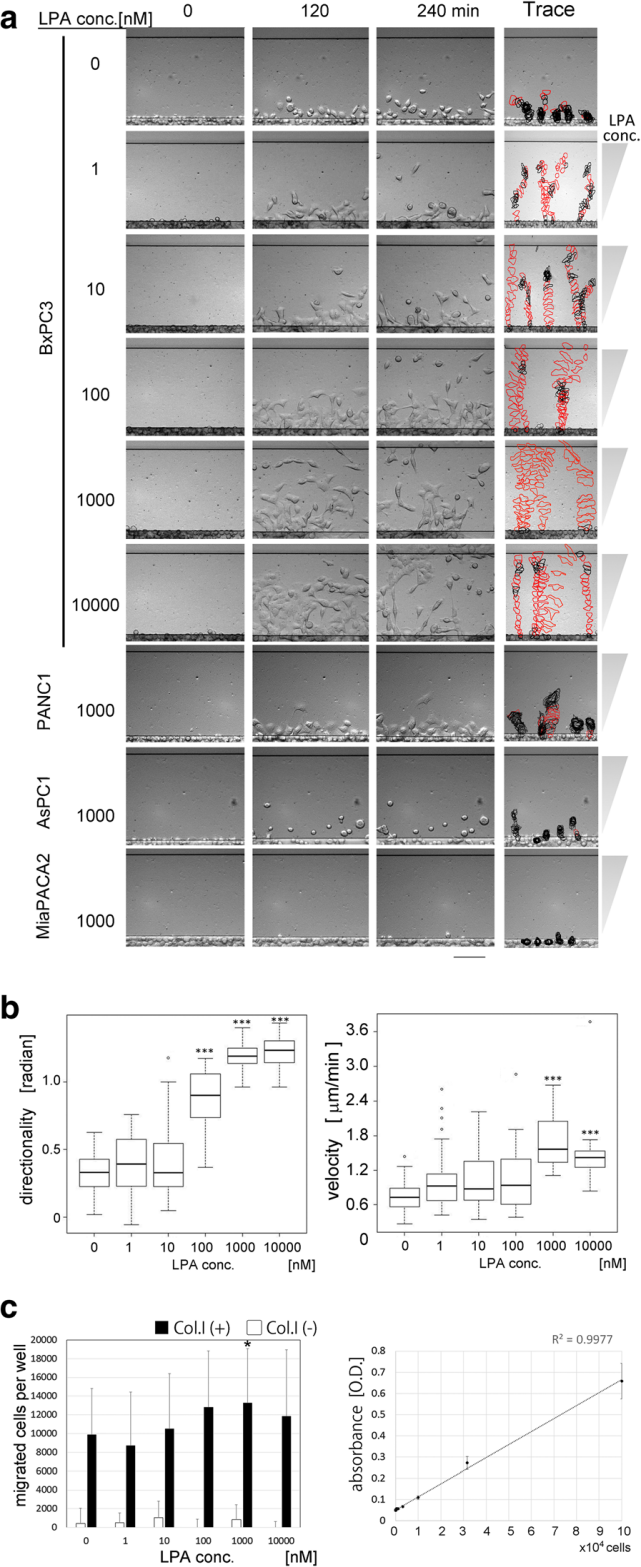
Chemotaxis of pancreatic cancer cells towards LPA detected by TAXIScan (**a** and **b**) or Boyden chamber (**c**). **a** Four pancreatic cancer cell lines were used for the TAXIScan method. Dose-dependency of BxPC3 chemotaxis towards LPA is observed. The migration images of PANC-1, AsPC1, and MIAPaCa-2 cells in the optimal conditions are also shown. Images taken at time 0, 120 and 240 min are shown. The morphologies of 4 or 5 representative migrating cells throughout the assays are shown on the “Trace” column. The outlines of the migrating cells were recorded every 10 min in this column. Cells migrating at more than 1 μm/min are shown in *red*. Data are representative of 3 independent experiments. Scale bar: 100 μm. **b** Quantitation of the directionality and velocity of migration of BxPC3 cells towards various concentrations of LPA. The graph on the left indicates the directionality and the graph on the right indicates velocity. *White circles* are outliers. Statistical analysis was done by the Kruskal-Wallis Test (Nonparametric ANOVA) followed by the Dunn’s Multiple Comparisons Test. Data are representative of 3 independent experiments. **c** Migration of BxPC3 cells towards LPA using Boyden chamber assay kit. The migrated cells were stained with the staining solution and the numbers of the migrated cells were estimated by measuring OD 560 nm based on the standard curve (*the graph on the right*). The assay results with the collagen I coated membrane (*black bar*) or the plain membrane (*white bar*) are shown in the graph on the left. Mean values of data are shown and the error bars represents the standard error (*n* = 6). Statistical analysis was conducted using the Student’s *t*-test. *p < 0.05 (vs. data without LPA)



LPA is known as a chemoattractant for cancer cells. To observe chemotactic migration of the pancreatic cancer cells towards LPA using the TAXIScan system, we used different concentrations of LPA to seek an optimal concentration for migration and observed that 1 μM of LPA was optimal for BxPC3 and PANC-1 cells (Fig. 2a) (An additional movie file shows this in more detail [see Additional file 4]). In the case of AsPC1 and MIAPaCa-2 cells, very few cells migrated towards LPA at the concentration ranging from 0.1 nM to 10 μM (only the 1 μM data is shown in Fig. 2a, an additional movie file shows this in more detail [see Additional file 5]).

**Additional file 4:** Chemotaxis of BxPC3 pancreatic cancer cells towards various concentrations of LPA on a collagen I coated coverslip. Images were taken every 5 min for 4 h and movies were created by TAXIScan Analyzer2 software. Representative of 3 independent experiments. Scale bar: 100 μm. (MP4 7612 kb)




**Additional file 5:** Chemotaxis of four kinds of pancreatic cancer cells towards 1 μM LPA on a collagen I coated coverslip. Images were taken every 5 min for 4 h and movies were created by TAXIScan Analyzer2 software. Representative of 3 independent experiments. Scale bar: 100 μm. (MP4 6258 kb)


BxPC3 cells were the most responsive to LPA of all the cell lines studied. Therefore, we quantitated the directionality and velocity of migration of BxPC3 cells in response to different concentrations of LPA. The directionality in response to LPA increased in a dose-dependent manner (Fig. 2b left panel). The velocity also increased in a dose-dependent manner in the dose range of 1 to 10 μM LPA (Fig. 2b right panel). These results were in agreement the TAXIScan images (Fig. 2a). We confirmed the same phenomenon by an existing assay method, the Boyden chamber method. In the Boyden chamber method, BxPC3 cells showed good response to LPA in a dose-dependent manner (Fig. 2c, left). The concentrations of LPA that elicited the migration of BxPC3 cells were observed to be similar in both methods.

### Expression of receptors for LPA on pancreatic cancer cells

**Fig. 3:**
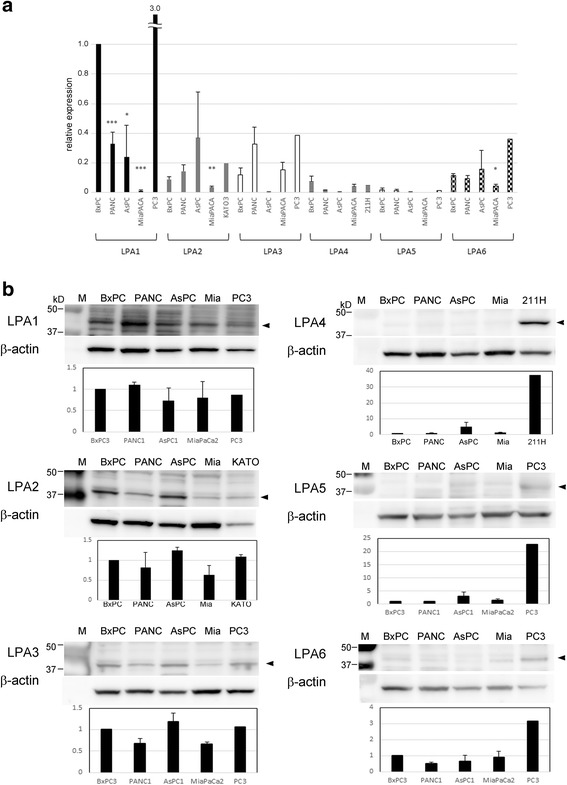
LPA receptor expression in pancreatic cancer cell lines. **a** mRNA expression in 4 pancreatic cancer cell lines determined by quantitative RT-PCR. The relative expression of each receptor was calculated based on the LPA_1_ expression in BxPC3. Data represent mean values of 3 independent experiments. The *error bars* represent the standard error. Statistical analysis was conducted using the Student’s *t*-test. *p < 0.05, **p < 0.01, ***p < 0.001 (vs. BxPC3). **b** Protein expression in 4 pancreatic cancer cell lines detected by SDS-PAGE and western blotting. A prostate cancer cell line, PC3, a gastric cancer cell line, KATOIII, and a pleural mesothelioma cell line, 211H, were used as positive controls. β-actin was used as a loading control and its expression is also shown. The *arrow-head* indicates the specific bands of each LPA receptor. M, protein marker; Mia, MIAPaCa-2; Photographs are representative of 3 independent experiments. The intensity of each band was measured and the relative expression of each receptor protein was calculated based on the receptor in BxPC3 cells. Quantitative data represent mean values of 3 independent experiments except the positive controls PC3 and 211H. The *error bars* represent the standard error

To confirm if the migration of cells was due to the LPA-dependent phenomenon, we evaluated the expression of LPA receptors. Because most published reports showed either only mRNA expression or only protein expression [12, 13, 27], we attempted to show both mRNA and protein expression systematically by using qRT-PCR and western blotting. As LPA_1_, LPA_2_, LPA_3_, LPA_4_, LPA_5_, and LPA_6_ are the known receptors for LPA; we used primers for these receptor isoforms (Additional file 1: Table S1) [27] to compare their mRNA expressions. In BxPC3 cells, based on the results of qRT-PCR, LPA_1_ was the most highly expressed receptor among all the 6 receptors (Fig. 3a), whereas LPA_2_, LPA_3_, and LPA_6_ were moderately expressed and LPA_5_ showed the lowest expression. In PANC-1 cells, LPA_1_ and LPA_3_ were the major receptors expressed. In AsPC1 cells, the mRNA expression of LPA_1_, LPA_2_, and LPA_6_ were detected, and in MIAPaCa-2 cells, the mRNA expression of most LPA receptors was extremely low. LPA_3_ expression was highest among the receptors for the MIAPaCa-2 cells (Fig. 3a).

We also evaluated the expression of these receptors at the protein level in the 4 pancreatic cell lines by western blotting using anti-LPA antibodies. All cell lines express a certain amount of LPA_1,_ LPA_2_ and LPA_3_ receptors, however, very low expression of LPA_4_, LPA_5_, and LPA_6_ receptors was observed in lysates of all cell lines compared to 211H, KATOIII or PC3 which were used as positive controls (Fig. 3b). The data from the migration assay and western blotting indicated that BxPC3 and PANC-1 cells express the LPA receptors and the migration images of the cells reflects the LPA-elicited migration.

### Signal transduction during migration of pancreatic cancer cells towards LPA

**Fig. 4:**
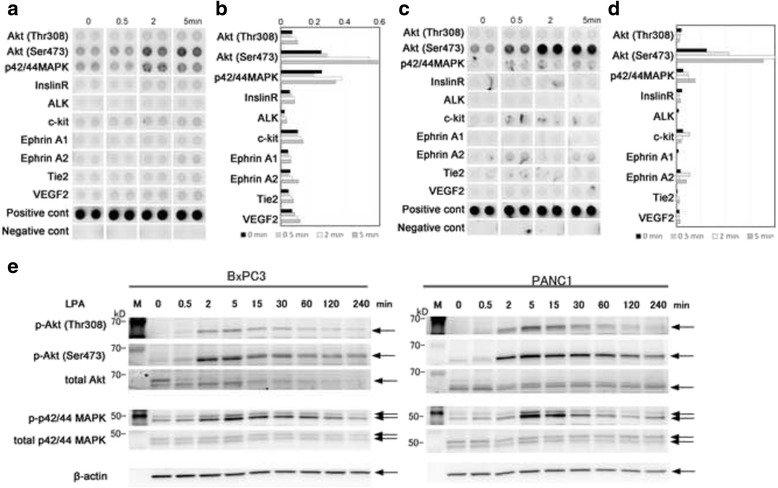
Phosphorylation of receptors or signaling molecules. **a** and **c** Images of phosphorylation of receptors in BxPC3 (**a**) or in PANC-1 (**c**) cell lines detected by Antibody Array. Data are representative of 3 independent experiments. **b** and **d** The quantitation of phosphorylation by measuring density of Antibody Array with BxPC3 data (**b**) or with PANC-1 data (**d**). **e** Phosphorylation of Akt or p44/42MAPK in BxPC3 and PANC-1 cell lines, as indicated. Cell lysates taken after LPA stimulation at each time point were analyzed by SDS-PAGE and western blotting. Anti-β-actin antibody was used as the internal control. *Arrows* indicate the specific band for each antibody. Data are representative of 2 independent experiments

**Fig. 5:**
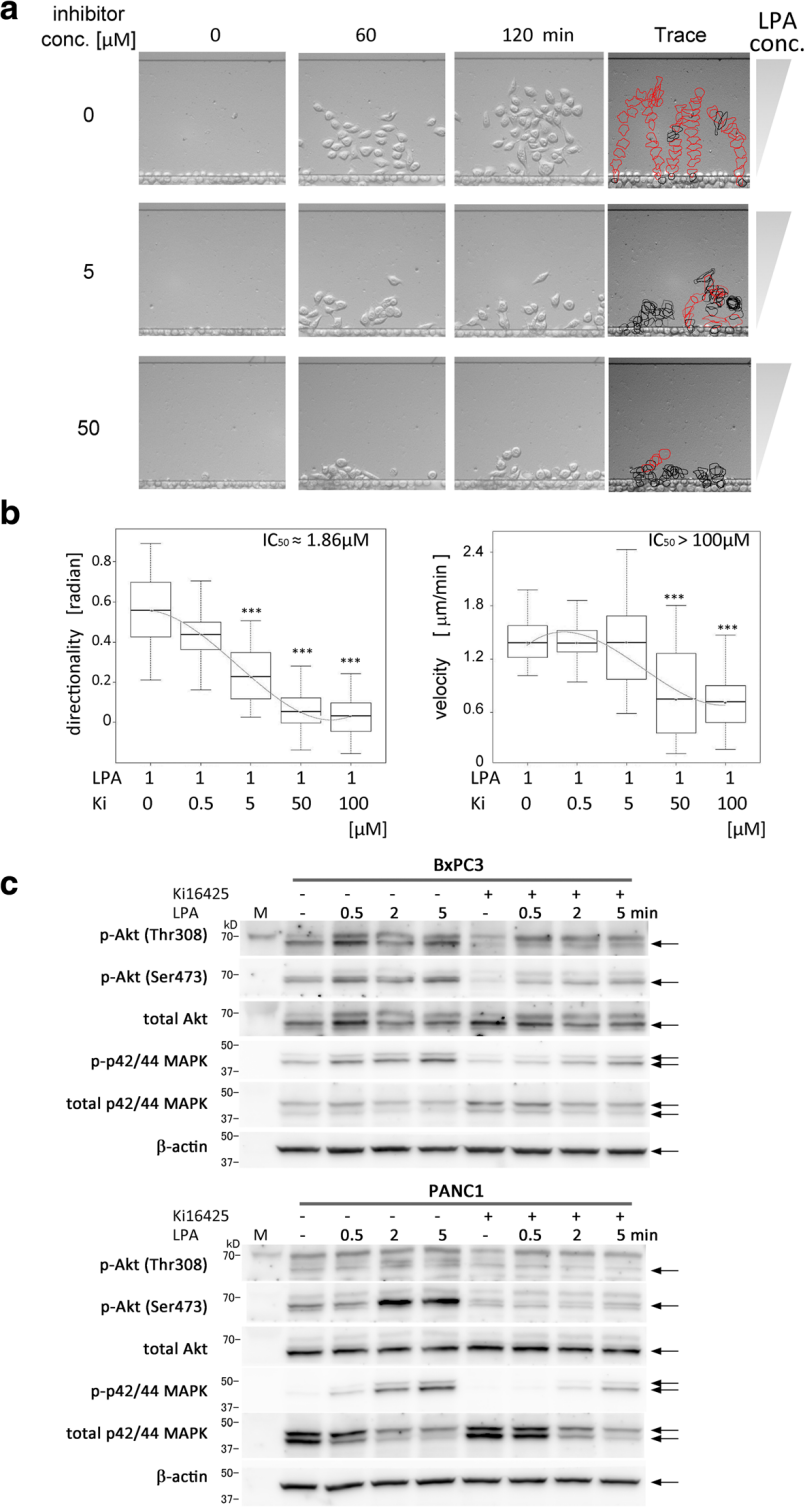
Inhibition of BxPC3 chemotaxis towards LPA by Ki16425. **a** BxPC3 chemotaxis towards 1 μM LPA with various concentrations of Ki16425. Cells were pre-incubated with Ki16425 for 24 h and the chemotaxis assay was performed using TAXIScan. Data are representative of 3 independent experiments. **b** Box-plots of the directionality and the velocity in BxPC3 migration towards LPA with Ki16425. The graph on the left indicates directionality and that on the right indicates velocity. The half maximal inhibitory concentration (IC_50_) values are also shown. Statistical analysis was done by the Kruskal-Wallis Test (Non-parametric ANOVA) followed by the Dunn’s Multiple Comparisons Test. ***p < 0.0001 (vs. data with 1 μM LPA and without Ki16425). Data are representative of 3 independent experiments. **c** Inhibition of phosphorylation of Akt or p44/42MAPK by Ki16425 in BxPC3 and PANC-1 cell lines, as indicated. Cell lysates taken after LPA stimulation at each time point were analyzed by SDS-PAGE and western blotting. Anti-β-actin antibody was used as the internal control. *Arrows* indicate the specific band for each antibody. Data are representative of 3 independent experiments

To further confirm that the migration was LPA-dependent, we determined phosphorylation of various molecules in BxPC3 and PANC-1 cells using the PathScan array, which enabled us to simultaneously evaluate the phosphorylation of 39 different molecules (Additional file 2: Table S2). We carried out phosphorylation assays at the time points 0.5, 2, and 5 min following LPA stimulation, due to uniform stimulation of cells by LPA on culture dishes, which precludes the use of an LPA concentration gradient similar to that of the TAXI Scan device. Using this array system, we observed that Akt (Thr308 and Ser473), p44/42MAPK, IRS-1, InsR, c-kit, EphA2, and Tie2 were phosphorylated after LPA stimulation in both BxPC3 (Fig. 4a, b) and PANC-1 cells (Fig. 4c, d). Of these phosphorylated proteins, Akt and MAPK are known to be key molecules involved in migration and proliferation. The phosphorylation of these signaling molecules after uniform stimulation was further observed by western blotting. The results obtained showed that Akt (Thr308 and Ser473), p44/42MAPK were phosphorylated after LPA stimulation, as expected, in both BxPC3 and PANC-1 cell lines within 5 min (Figs. 4e and 5c). For the record, we also checked longer time points, such as 15, 30, 60, 120, and 240 min which were similar to the time points used in the TAXIScan experiments, but no additional increase in phosphorylation of these molecules was observed (Fig. 4e). These data further support the establishment of the assay system of cancer cell migration towards LPA.

### Effect of inhibitor on migration towards LPA

We also tested the effect of an LPA inhibitor, Ki16425 [28], on LPA-elicited migration of BxPC3 cells. When the cells were treated with Ki16425, the migration of the cells towards LPA was abrogated in a dose-dependent manner (Fig. 5a, b, an additional movie file shows this in more detail [see Additional file 6]). The half maximal inhibitory concentration (IC_50_) value for directionality was ≈ 1.86 μM (Fig. 5b, left graph). Owing to weak inhibition of velocity by Ki16425, the IC_50_ value for velocity was >100 μM (Fig. 5b, right graph). When the cells were treated 50 μM Ki16425, the phosphorylation of Akt and MAPK was reduced, as observed during western blot analysis (Fig. 5c). The pancreatic cancer cells showed LPA-elicited chemotactic migration with clarity in the TAXIScan chamber, and this phenomenon was vigorously supported by the inhibition of the intracellular signaling with Ki16425.



**Additional file 6:** Inhibition of BxPC3 chemotaxis towards LPA by Ki16425. Cells were pre-incubated with Ki16425 for 24 h and the chemotaxis assay towards 1 μM LPA was performed using TAXIScan. Images were taken every 5 min for 4 h and movies were created by TAXIScan Analyzer2 software. Representative of 3 independent experiments. Scale bar: 100 μm. (MP4 6072 kb)


## Discussion

In this study, we established a pancreatic cancer cell migration assay system by using the TAXIScan device. We found that coating of scaffolds such as collagen and Matrigel on glass, similar to that in some published studies using other methods, was necessary for successful adhesion and migration. BxPC3 and PANC-1 cells migrated towards LPA in a dose-dependent manner, which was clearly inhibited by an LPA inhibitor, Ki16425. This is the first report of pancreatic cancer cell migration monitored by the TAXIScan system that enables analysis of multiple parameters, including directionality, velocity, and cell morphology. Additionally, this is the first report simultaneously comparing the TAXIScan and Boyden chamber methods. The Boyden chamber method has been used for over 50 years [29], the limitations of this method have been pointed out by several researchers. In this method, a membrane of 10 μm thickness, having holes of 8 μm diameter (in this study) with random density, separates the upper and lower wells (see Additional file 7). It is thought that cells are able to sense differences in the chemoattractant concentration between these two wells. Although this method appears simple, it has certain limitations. (I) The density of holes may not be uniform. (II) The micro-structure inside the hole, e.g., a micro-channel of 10 μm length × 8 μm diameter, is unknown, and the chemoattractant gradient is not measurable. (III) A large number of cells is necessary for this assay (1.5 × 10^5^ cells per well in this study). (IV) A considerable amount of chemoattractant is necessary (500 μL per well in this study), which is expensive. (V) The process of cell migration is not visible. (VI) The device only displays the numbers of migrated cells. (VII) The obtained data may have high background noise. (VIII) The density of cells migrating to the lower side of the membrane is not uniform. A few advantage of this method are as follows: (I) It has a simple structure; (II) the apparatus itself (without coating materials) is inexpensive; and (III) it is well known and widely used. On the other hands, the advantages of TAXIScan are as follows [14] (see also Additional file 8): (I) it has an uniform micro-channel (260 μm length × 1000 μm width × 8 μm height); (II) the chemoattractant, which is placed on one end of the micro-channel, defuses uniformly through the channel, resulting in a stable concentration gradient [14]; (III) a small number of cells is required for analysis (100 or less cells per channel); (IV) a small and inexpensive amount of chemoattractant is necessary (1 μL per channel); (V) migrating cells are observable; (VI) images obtained during migration are recorded automatically; (VII) data obtained from this assay including that on morphology, behavior, directionality, and velocity, are more informative. However, some demerits of TAXIScan are as follows: (I) although the running cost is low, the initial cost is high, and (II) it is not well-known yet. In fact, it may not be appropriate to position TAXIScan as an alternate to the Boyden method, because both methods utilize completely different equipment and data collection methods, and the quality of data obtained using these methods is entirely different (Additional files 7 and 8). However, because of lower requirement of samples and the collection of more informative data, the approach to cancer cell migration using TAXIScan is more useful than analysis using existing techniques such as the Boyden chamber method. With the TAXIScan system, the characteristics of pancreatic cancer cells can be analyzed in detail. Moreover, our system can be adopted for migration studies in other types of cancer cells.

In the Boyden chamber method, a certain number of cells without LPA was observed to migrate, indicating a high background (Fig. 2c), similar to that reported previously [30–33]. This high background with the Boyden chamber method is considered to be due to the thickness of the membrane (10 μm in this study). In TAXIScan method, cells without LPA were observed to migrate for more than 10 μm (up to 100 μm) (Fig. 2a), explaining this phenomenon. From this point of view, we could argue that TAXIScan has a wider dynamic range to detect cell migration.

Herein, 4 pancreatic cancer cell lines were analyzed and only 2 of these cell-lines, BxPC3 and PANC-1, showed good migration towards LPA with reasonable co-evidence on the expression of LPA receptors. The reason why AsPC1 and MIAPaCa-2 cells do not migrate towards LPA is still unknown. BxPC3 and PANC-1 do express LPA_1_, LPA_2_, and LPA_3_; however, these cell lines do not express LPA_4_, LPA_5_, and LPA_6_ as observed during western blotting (Fig. 3b). The latter 3 receptors are likely not involved in cell migration but might be involved in other cellular functions.

LPA inhibitor, Ki16425, shown in this study is believed to block human LPA_1_ and LPA_3_ receptors [28]; 10 μM of Ki16425 significantly blocked the migration of cancer cells [13]. In our system, Ki16425 clearly inhibited BxPC3 cell migration towards LPA at 5-50 μM concentrations, indicating that TAXIScan and BxPC3 cells are the best tools for screening inhibitors of pancreatic cell migration. Utilizing such a new method, new molecules for regulating pancreatic cancer metastasis can be identified, and the limited treatment options and the poor prognosis of this disease can be overcome. Studies on neutrophils have tested various kinds of compounds and found that some compounds inhibit neutrophil function, leading to the successful selection of several effective molecules [34]. Collectively, it can be concluded that the system established in our study can be a powerful tool for cancer research and drug discovery in seeking effectors and inhibitors for analyzing cancer cell function. We are currently looking for and screening such molecules that can regulate pancreatic cancer cell migration; some promising molecules will be reported in the near future.

## Conclusions

We established a novel pancreatic cancer cell migration assay system that provides optical and quantitative information simultaneously. Using this system, we demonstrated that BxPC3 and PANC-1 cells showed good migration towards LPA. The effect of an LPA inhibitor, Ki16425, was detected clearly in this system, which was confirmed by the reduction in the phosphorylation of signal transduction molecules, Akt and MAPK. As this method provides a large amount of information on migrating cells simultaneously, such as their morphology, directionality, and velocity, with a small volume of sample, it can be a powerful tool for analyzing the characteristics of cancer cells and for evaluating factors affecting cellular functions.

## Additional files

Additional file 1: Table S1. Primers used for the quantitative RT-PCR. Total 6 pairs of primers for LPA receptors (LPA1, LPA2, LPA3, LPA4, LPA 5, and LPA6) were used for this study, based on the information reported previously (27). (DOCX 14 kb)

Additional file 2: Table S2. Targets for PathScan RTK signaling array. The phosphorylation of 39 different molecules in BxPC3 and PANC-1 cells was evaluated using the PathScan array. Details are described in Methods section. (DOCX 14 kb)

Additional file 7: The modified Boyden chamber assay. A) Schematic diagram (sagittal section) of one well of the modified Boyden chamber assay (Transwell). Cells in the chemotaxis buffer are located in the upper chamber and the chemoattractant the chemotaxis buffer is added to the lower chamber. B) Schematic diagram of the membrane part of the modified Boyden chamber. The membrane separates the upper and the lower chamber. The matrix is coated on the lower side of the membrane. C) Photographs of the lower side of the membrane after the assay. Cells are stained with the staining solution accompanied with the assay kit. Magnification: 400×. (TIFF 98113 kb)

Additional file 8: The TAXIScan assay. A) Schematic diagram (sagittal section) of one channel of the TAXIScan chamber. The chamber is filled with the chemotaxis buffer (light brown color). Cells are located on the one side of the micro-channel and the chemoattractant (red color) is placed on the other side of the micro-channel. B) Schematic diagram (sagittal section) of the micro-channel. The chemoattractant is defused in the micro-channel, which forms the stable concentration gradient. Cells on the matrix-coated coverslip migrates towards the gradient of the chemoattractant in the micro-channel. C) Photograph of cells migrating towards the chemoattractant. The image is taken from underneath of the TAXIScan chamber. (TIFF 98112 kb)

## Abbreviations

BSA: Bovine serum albumin
EDTA: Ethylenediamine tetraacetic acid
EGF: Epidermal growth factor
Eph: Ephrin
FBS: Fetal bovine serum
IC50: The half maximal inhibitory concentration
InsR: Insulin receptor
IRS-1: Insulin receptor substrate 1
LPA: Lysophosphatidic acid
MAPK: Mitogen-activated protein kinase
RTK: Receptor tyrosine kinase
SDS-PAGE: Sodium dodecyl sulfate-polyacrylamide gel electrophoresis
Tie2: Tyrosine kinase with Ig-like loops and epidermal growth factor homology domains-2
