# Supplementary material for: Evaluation of pancreatic cancer cell migration with multiple parameters in vitro by using an optical real-time cell mobility assay device
Source: BMC Cancer. 2017 Mar 31;17:234. doi: 10.1186/s12885-017-3218-4 (PMC5374612; doi:10.1186/s12885-017-3218-4)
Supplement: Supplementary file 5 — Primers used for the quantitative RT-PCR. Total 6 pairs of primers for LPA receptors (LPA1, LPA2, LPA3, LPA4, LPA 5, and LPA6) were used for this study, based on the information reported previously (27). (DOCX 14 kb) [file 12885_2017_3218_MOESM1_ESM.docx]

**Table S1**. Primers used for the quantitative RT-PCR.

Total 6 pairs of primers for LPA receptors (LPA1, LPA2, LPA3, LPA4, LPA 5, and LPA6) were used for this study, based on the information reported previoudly (27).

|  |  |  |
| --- | --- | --- |
|  | Forward | Reverse |
|  |  |  |
| LPA1 | AATCGGGATACCATGATGAGTCTT | CCAGGAGTCCAGCAGATGATAAA |
| LPA2 | CGCTCAGCCTGGTCAAGACT | TTGCAGGACTCACAGCCTAAAC |
| LPA3 | AGGACACCCATGAAGCTAATGAA | GCCGTCGAGGAGCAGAAC |
| LPA4 | CCTAGTCCTCAGTGGCGGTATT | CCTTCAAAGCAGGTGGTGGTT |
| LPA5 | CGCAATGGCATGTGTGTTC | TCCACGCTGGCTGTATATGG |
| LPA6 | AAACTGGTCTGTCAGGAGAAGT | CAGGCAGCAGATTCATTGTCA |
| βactin | ACGAGGCCCAGAGCAAGAG | GACGATGCCGTGCTCGAT |
|  |  |  |
|  |  |  |
