# Supplementary material for: Evaluation of pancreatic cancer cell migration with multiple parameters in vitro by using an optical real-time cell mobility assay device
Source: BMC Cancer. 2017 Mar 31;17:234. doi: 10.1186/s12885-017-3218-4 (PMC5374612; doi:10.1186/s12885-017-3218-4)
Supplement: Supplementary file 6 — Targets for PathScan RTK signaling array. The phosphorylation of 39 different molecules in BxPC3 and PANC-1 cells was evaluated using the PathScan array. Details are described in Methods section. (DOCX 14 kb) [file 12885_2017_3218_MOESM2_ESM.docx]

**Table S2**. Targets for PathScan RTK signaling array.

The phosphorylation of 39 different molecules in BxPC3 and PANC-1 cells was evaluated using the PathScan array. Details are described in Methods section.

|  |  |  |
| --- | --- | --- |
| Receptor Tyrosine Kinases |  | Signaling molecules |
| EGFR/ErbB1 |  | Akt/PKB/Rac (Thr308) |
| HER2/ErbB2 |  | Akt/PKB/Rac (Ser473) |
| HER3/ErbB3 |  | p44/42MAPK (ERK1/2) |
| FGFR1 |  | S6 Ribosomal Protein |
| FGFR3 |  | c-Abl |
| FGFR4 |  | IRS-1 |
| InsR |  | Zap-70 |
| IGF-IR |  | Src |
| TrkA/NTRK1 |  | Lck |
| TrkB/NTRK2 |  | Stat1 |
| Met/HGFR |  | Stat3 |
| Ron/MST1R |  |  |
| Ret |  |  |
| ALK |  |  |
| PDGFR |  |  |
| c-kit/SCFR |  |  |
| FLT3/Flk2 |  |  |
| M-CSF/CSF-1R |  |  |
| EphA1 |  |  |
| EphA2 |  |  |
| EphA3 |  |  |
| EphB1 |  |  |
| EphB3 |  |  |
| EphB4 |  |  |
| Tyro3/Dtk |  |  |
| Axl |  |  |
| Tie2/TEK |  |  |
| VEGFR2/KDR |  |  |
|  |  |  |
